# Effect of Different Individualised Strength Training Approaches to Improve Physical Performance in Male Basketball Players

**DOI:** 10.3390/sports13070214

**Published:** 2025-07-02

**Authors:** Francisco J. Barrera-Domínguez, Bartolomé J. Almagro, Jorge Molina-López

**Affiliations:** Faculty of Education, Psychology and Sport Sciences, COIDESO, University of Huelva, 21007 Huelva, Spain; bartolome.almagro@dempc.uhu.es (B.J.A.); jorge.molina@ddi.uhu.es (J.M.-L.)

**Keywords:** acceleration, deceleration, plyometric, agility, team sports

## Abstract

Training individualisation is a key principle for maximising improvements in players’ performance, but there are still few approaches to individualisation of training in basketball players. The aims of this study were as follows: (I) to analyse the effects of two individualised training approaches on performance in male basketball players; (II) to compare the inter-individual differences in adaptations of these physical abilities; and (III) to assess differences in physical performance between two time periods of training intervention. Forty-five male basketball players (age, 22.3 ± 4.18 years; body height, 1.86 ± 0.15 m; body mass, 86.3 ± 7.85 kg) were divided into three groups: a vertical group (n = 15), who performed an individualised training programme based on a force–velocity profile; a horizontal group (n = 15), who underwent individualised intervention based on change of direction deficit; and a control group (n = 15). The assessments included jump and speed tests. Strength training was administered twice weekly for 8 weeks. Both intervention groups demonstrated large significant group x time interactions in jump (η_p_^2^ ≥ 0.24, *p* < 0.01) and speed (η_p_^2^ = 0.23, *p* < 0.01), with no significant changes in the control. The largest performance gains were achieved in the specific force orientation targeted by each intervention. These findings suggest that addressing the individual needs of each player and the specificity of the physical ability are key considerations for training programming in basketball players.

## 1. Introduction

Basketball is a team sport of an unpredictable, multidirectional, and intermittent nature characterised by a combination of high-intensity, short-duration actions with low to medium intensity movements [1]. However, high-intensity ballistic actions such as jumps, accelerations, decelerations, and changes in direction (COD) are key performance indicators for basketball players and the ones that determine the decisive actions in real game situations [2]. In this regard, basketball players perform maximal-intensity multidirectional actions every 1 to 3 s during a game [1], with deceleration actions involving COD being executed most frequently due to sport-specific demands [3]. Notably, 15.1% of CODs in a game are executed at high intensities (<−3.5 m·s^−2^) [4]. Accelerations and linear speed (LS) are also considered key abilities, with basketball players performing over 105 sprints per game within short distances between 3.9 and 9.5 m, which represent 2–6% of total game time [5]. Jumping is similarly essential, as it is a skill performed in most technical actions, both offensive (e.g., jump shots or layups) and defensive (e.g., blocks); moreover, during a competition, male players perform between 41 and 56 jumps [5]. Therefore, strength and conditioning basketball coaches must focus on developing accurate and effective training programmes to create fast, efficient, and robust basketball players to execute jumps, accelerations, and decelerations at maximum intensity in all directions [6].

The performance of these high-intensity, multidirectional ballistic actions is closely linked to the ability of each player to mobilise their own body mass, both as much as possible and within the shortest time possible [7]. Given the complexity and multidirectionality of these ballistic actions with high demands for fast force (<400 ms) [6], many studies highlight lower-limb neuromuscular power and rate of force development as critical factors for effective performance [8,9]. Numerous training methodologies have been evaluated for their impact on ballistic performance [10], with plyometric training being the most extensively studied approach. This method has shown improvements in ballistic performance, with effect sizes (ES) from small (ES = 0.26) to large (ES = 2.8), depending on the test used or the sample characteristics assessed [11]. Specifically, plyometric training yields significant performance gains in basketball players due to the specificity to the sport itself [12]. However, a recent meta-analysis examining the effect of different training proposals on the performance of COD actions in adult basketball players [13] revealed that, to date, there is no intervention in basketball players that adheres to one of the most important training principles: individualisation [14]. Furthermore, it was suggested that training without considering individual needs or responses to training is not efficient for improving ballistic performance in basketball players [13]. Therefore, individualised training approaches are recommended to improve performance in basketball players.

New paradigms that have been developing in recent years advocate for the individualisation of training and specify that high-intensity ballistic performance is largely influenced by each players’ individual force and velocity needs, both with vertical [15] and horizontal force orientations [16]. On the one hand, Samozino et al. [17] confirmed that each player has an optimal vertical-force–velocity (F-V) profile that maximises ballistic movements by achieving an ideal balance of force and velocity in vertical ballistic actions [7]. On the other hand, Barrera-Domínguez et al. [16] examined the effect of COD Deficit (CODD) on players’ linear and multidirectional performance, determining optimal thresholds for different cutting angles at which players achieved their best performance across the whole linear and multidirectional speed profile. Players with higher performance in LS actions are often inefficient in COD, because they reach a high sprint momentum that is difficult to manage over the deceleration phase and the cutting action itself [18]. Conversely, players with higher performance in COD actions exhibited lower performance in LS due to being less efficient at accelerating [16]. Given the multidirectional nature of basketball, it is advisable to seek a trade-off between LS and COD for each individual player in order to create robust three-dimensional players adapted to the sport’s demands [16].

The existing literature reveals that both approaches attempt to improve sport performance by seeking the optimal balance between individual force and velocity needs in different sport-specific movements, such as jumping through the F-V profile and multidirectional speed through the CODD. However, to the best of the authors’ knowledge, there is no longitudinal study that analyses and compares the effects of both approaches on the performance of basketball players. Thus, firstly, the main purpose of this study was to analyse the effects of two individualised training approaches, one based on the F-V profile, and the other based on CODD, on the performance of male basketball players. Secondly, this study aimed to compare the inter-individual differences in adaptations of these physical abilities. Finally, we aimed to assess the differences in physical performance between two different time periods (four and eight weeks of training intervention). As both individualised training approaches seek to align a balance between the individual force and velocity demands of each player, it was initially hypothesised that both approaches would be effective and produce increases in player performance, but these effects would probably be specific to the force orientation trained.

## 2. Materials and Methods

### 2.1. Participants

Forty-five male basketball players from three teams belonging to the same competitive level (i.e., Spanish N1 National League) were selected non-probabilistically by convenience and classified into three groups, as follows: a vertical group (VG, n = 15; age, 22.9 ± 6.52 years; height, 1.88 ± 0.08 m; body mass, 90.3 ± 23.0 kg), who underwent an individualised F-V profile intervention following the protocol of Jimenez-Reyes et al. [19]; a horizontal group (HG, n = 15; age, 21.1 ± 1.45 years; height, 1.86 ± 0.07 m; weight, 85.8 ± 8.91 kg), who underwent individualised intervention based on the CODD threshold [16]; and a control group (CG, n = 15; age, 22.7 ± 4.93 years; height, 1.84 ± 0.05 m; body mass, 82.2 ± 6.00 kg), in which no specific, nor individualised, resistance intervention was carried out. All players had at least six years of prior experience playing at the federated level and one year of strength training directly prior to the intervention; they carried out regular training four days per week during the competitive period and did not present any injury six months prior to or during the investigation; they did not perform any additional resistance training beyond that prescribed; and all of them completed 100% of the programmed intervention. This research was developed throughout the second part of the season and finished just before the end of the competitive period. All players were informed about the study’s objectives and gave their written consent to participate in it. The experimental protocol adhered to the latest version of the Declaration of Helsinki, and it was approved by the Andalusian Ethics Committee for Biomedical Research (reference number: FBD_UHU2020; approval date: 8 October 2020).

### 2.2. Study Design and Procedures

**Figure 1:**
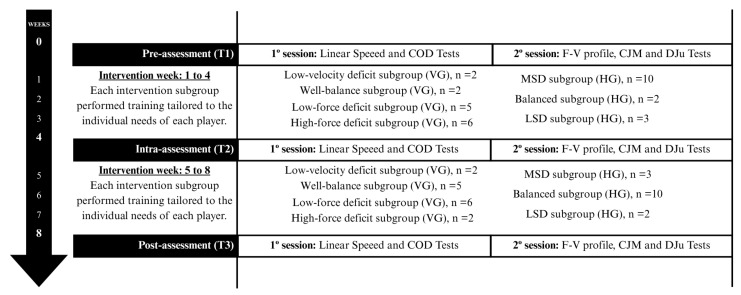
Diagram illustrating the course of the research experiment. COD, change of direction; F-V, force–velocity; CMJ, countermovement jump; DJu, unilateral drop jump; VG, vertical group; HG, horizontal group.

A quasi-experimental repeated measure, 8-week follow-up design was used, in which all groups performed four on-court basketball training sessions (90 min/session), and one game (40 min/game) per week. Moreover, both intervention groups undertook two strength and conditioning sessions (60 min/session) every week. The intervention design included three assessment points: a pre-assessment (T_1_) conducted one week prior to the intervention; an intra-intervention evaluation (T_2_), at week 4 (just in the middle of the intervention), in which the training sessions were readjusted based on the new assessment data; and a post-evaluation (T_3_) just at the end of the intervention (Figure 1).

One week before the study began, the players completed two familiarisation sessions with the testing procedures. For T_1_, T_2_, and T_3_ assessments, the evaluations were organised into two sessions, always before the sports training itself (7–8 pm) with a 48 h interval between sessions. In the first session, horizontal-force-oriented tests were included, as follows: (i) LS and COD tests. In the second session, vertical-force-oriented tests were evaluated, as follows: (ii) F-V profile, countermovement jump (CMJ), and unilateral drop jump (DJu). All tests were carried out on the hardwood court where the players regularly trained and played. The players were instructed to attend the assessment well hydrated, adequately rested, and to avoid any intense physical activity during the preceding 24 h. Additionally, they were asked to refrain from consuming caffeine, food (for the preceding 3 h), and alcohol (for at least 24 h prior to testing). Before each assessment, the players completed a standardised warm-up consisting of 5 min of low-intensity ball handling, 5 min of hip mobility and core activation exercises, 5 min of landing, multidirectional bounding, and acceleration–deceleration drills, followed by 5 min of gradual intensity increases to prepare for the specific tests. Each player performed 3 attempts for each test, with a 2-minute rest interval between attempts. The average of all attempts for each test was used for further analysis.

### 2.3. Training Intervention

Both intervention groups carried out their strength training twice weekly, 60 min before the on-court basketball session. The workload was equalised and adjusted following previous scientific recommendations [19,20] by performing 18 sets per week, divided into 6 different exercises (3 sets/exercise), and a 120 s rest period was performed between sets to optimise intra-session recovery (detailed in Tables S1 and S2). Throughout the intervention, the players were verbally instructed and encouraged to perform each exercise at maximum intensity [21].

For the VG, the players were classified according to the F-V profile, as follows: “well-balanced”, if they had less than 10% imbalance from the optimal F-V profile (well-balanced subgroup VG, n = 2); “low deficit”, when they had between 10 and 40% (low-force deficit subgroup VG, n = 5; low-velocity deficit subgroup VG, n = 2); and “high deficit”, when they exceeded a 40% imbalance (high-force deficit subgroup VG, n = 6) [19]. Training for the VG was then individualised according to this classification, targeting specific velocity zones within the F-V profile to match each player’s individual needs (Table S1).

On the other hand, the HG players were classified according to the CODD180°. The HG players were labelled as follows: “multidirectional speed dominant” (MSD; HG, n = 10), if they had less than 43% CODD180°; “balanced”, when they had between 43 and 49% (HG, n = 2); and “linear speed dominant” (LSD; HG, n = 3), when they exceed 49% CODD180° [16]. As basketball is a multidirectional sport, in which players must be able to execute both LS and COD up to 180° at maximum intensity [22], the HG training programme was individualised to create more efficient players in COD by seeking the trade-off between LS and COD according to the individual needs of each player. Table S2 details the intervention performed on each HG subgroup.

### 2.4. Physical Performance Test

Vertical Test Assessments: countermovement jump, unilateral drop jump, and vertical-force–velocity profile. The vertical tests were evaluated using a Chronojump contact platform (Chronojump Boscosystem^®^, Barcelona, Spain) [23]. Each subject performed a DJu, a CMJ with body weight, plus 5 CMJs with a trap bar and additional loads ranging from 20 to 60 kg in a random order. The CMJ tests, with and without extra load, were performed bilaterally, while DJus were unilateral. The DJu was performed from an initial height of 30 cm [24], and each subject dropped down until they came into contact with the platform with only one leg, in order to jump as high as possible, finally landing with the same leg. Each DJu and CMJ attempt was considered valid if both hands were placed on the hips for DJu and CMJ without extra load, or on the trap bar with elbows extended for loaded CMJ, the knees were not bent during the jump flight time, and whether the landing was made at the same point as the take-off. For the loaded CMJ to be valid, it had to reach at least 10 cm in height [25].

An Excel spreadsheet downloaded from www.jbmorin.net (JUMPFVPprofile(1).xlsx) was used to apply Samozino et al.’s [7] method and obtain the theoretical maximum values of force (F0), velocity (V0), and power (Pmax). Additionally, the spreadsheet also showed the individual optimal theoretical F-V profile for each subject and the F-V imbalance of each individual with respect to the F-V profile [17]. In addition, the values of the reactive strength index (RSI) were calculated through the flight time/contact time ratio for each leg extracted from the DJu.

Speed Test Assessments: Linear and change of direction speed. The times in the speed tests were measured with single-beam photocells (Chronojump Boscosystem^®^, Barcelona, Spain) [16]. The photocells were placed 2 m apart from each other and at a height of 1.10 m (approximately the height of the players’ hips). Each player was placed 0.5 m behind the first gate in a two-point split stand. Then, each player accelerated at maximum speed to the second gate. The speed tests were as follows: (I) an LS test, in which the photocells were placed 10 m away in a straight line; and (II) a COD test, in which each player ran 5 m to a pivot point marked on the floor and then changed direction at 45° (COD45°), 90° (COD90°), and 180° (COD180°) to go to the second gate in the shortest possible time. The COD tests were performed on both sides, with laterality defined by the leg on which the players leaned when performing the COD mechanics [26]. The basketball players’ mean speed performance was calculated as the average of the four speed tests assessed, and the CODD for each angulation was obtained as follows [27]:

$$CODD\%=\frac{COD\;time-LS\;time}{LS\;time}\times 100$$

### 2.5. Statistical Analysis

Data were expressed as the mean and standard deviation. The normality of the data were tested and confirmed using the Shapiro–Wilk test (*p* > 0.05). Homoscedasticity was determined with the Levene test. The relative and absolute reliability of the tests was evaluated by the intraclass correlation coefficient (ICC) and the coefficient of variation (CV) for all assessments. An ICC ≥ 0.8 was considered strong and a CV < 5% was established as an intra-day reliability criterion. Training effects were evaluated using a 3 (groups: CG, VG, and HG) × 3 (time: T_1_, T_2_, and T_3_) repeated measures ANOVA model, and the effect size (ES) were determined using the partial eta-square (η_p_^2^). ES was considered trivial (<0.003), small (0.01), moderate (0.06), or large (>0.14) [28]. The model was adjusted by inter-group differences, T1. Additionally, the Bonferroni post hoc test for multiple comparisons was performed to identify where significant changes occurred, and the ES was determined using the Cohen’s d. ES was considered trivial (<0.10), small (0.10–0.39), moderate (0.40–0.79), or large (>0.80) [28]. Finally, responders (Rs) and non-responders (NRs) to the interventions were determined through calculating 2CV [29]. The players were labelled as NRs if they did not achieve a post-intervention performance increase greater than 2CV, and this was established as follows: CMJ: 1.452 cm, DJu: 1.086 cm, RSI: 0.084 cm, LS: 0.068 s, COD45°: 0.082 s, COD90°: 0.060 s, and COD180°: 0.092 s, respectively. The alpha level was set to 0.05. Statistical analysis was performed with SPSS version 25 for Windows (SPSS, Inc., Chicago, IL, USA).

## 3. Results

Table 1 shows the baseline performance, inter-group differences, and reliability metrics (ICC and CV values). There were no significant differences between the groups at the baseline for any variables, except for RSI (*p* ≤ 0.01), with differences mainly between the VG and HG. Relative and absolute reliability measures were confirmed for all assessments (ICC ≥ 0.90; CV ≤ 4.94).

Table 2 shows a comparative analysis of inter- and intra-group changes in classification and performance variables. According to the classification variables, the F-V profile imbalance showed large significant group × time interactions (η_p_^2^ = 0.25, *p* < 0.01), with the VG players achieving the best F-V profile after 8 weeks of intervention. Concerning the classification variable used to individualise HG training, CODD180°, large group × time changes were observed (η_p_^2^ = 0.17, *p* = 0.08), with the HG being the only group in which all players reached a balanced CODD180° and the one that showed the greatest significant differences between the CG (d = –1.88, *p* < 0.01) and VG (d = –1.20, *p* < 0.05) after 8 weeks of training.

On the other hand, these observed changes in the classification variables used to individualise training resulted in different magnitudes of changes in the players’ performance variables. In this regard, both the jump (η_p_^2^ ≥ 0.24, *p* < 0.01) and speed (η_p_^2^ = 0.23, *p* < 0.01) variables showed large significant group x time interactions. A post hoc Bonferroni analysis revealed that, although both training approaches improved vertical jump and showed significant differences compared to the CG, the VG achieved the largest improvements at both 4 weeks (≥7.29%, d ≥ 1.47, *p* < 0.01) and 8 weeks (≥17.0%, d ≥ 3.70, *p* < 0.01) of training. Both individualised intervention groups also showed improvements in speed performance, though the HG achieved the greatest improvements (≥5.49%, d ≥ 2.04, *p* < 0.01) after 8 weeks of training.

**Figure 2:**
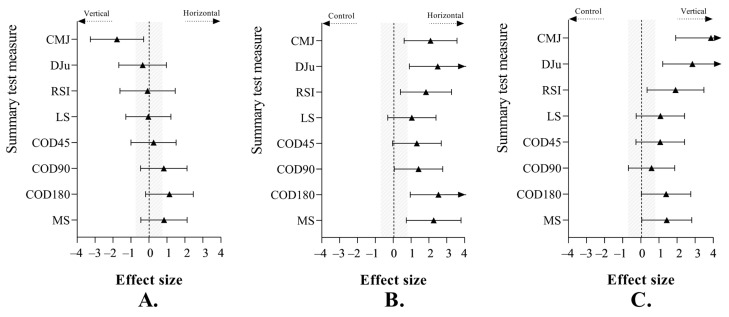
Standardised differences (Cohen’s d, CI95%) for countermovement jump (CMJ), unilateral drop jump (DJu), reactive strength index (RSI), 10 m linear sprint (LS), change of direction at 45° (COD45°), 90° (COD90°), and 180° (COD180°), and mean speed (MS) time according to (**A**) vertical group (VG) vs. horizontal group (HG); (**B**) control group (CG) vs. HG; (**C**) CG vs. VG.

The results of the inter-group analysis for post-assessment changes in performance variables are illustrated in Figure 2. Standardised differences for jump and speed outcomes showed significantly better results in the VG (d ≥ 1.43, *p* < 0.01) and HG (d ≥ 1.42, *p* < 0.01) compared to those of the CG at the post-test stage. Likewise, large significant differences were found between the HG and VG only in CMJ (d = −1.78, *p* < 0.01).

Finally, Table 3 shows the percentage of NRs to each individualised training intervention. The non-individualised training in the CG proved to be inefficient in producing changes in most players, with up to 87% of NRs. Each individualised training group only obtained 22% of NRs. The COD90° variable showed the highest number of NRs, regardless of the training group.

## 4. Discussion

The current study aimed (I) to analyse the effects of two individualised training approaches, one based on F-V profile and the other based on CODD, on the ability of male basketball players to jump, accelerate, and change direction; (II) to compare the inter-individual differences in adaptations of these physical abilities; and (III) to assess differences in physical performance between two different time periods (four and eight weeks of training intervention). The most notable findings of this study were as follows: (A) each of the individualised training programmes was effective in improving the variable on which the training was based. That is, the VG and HG were more efficient in the F-V profile and CODD180°, respectively. Additionally, (B) both individualised training approaches led to performance improvements across all measured outcomes after 8 weeks. (C) These improvements were of greater magnitude in actions specific to the force orientation training focus; therefore, greater gains were observed in vertical variables among players who underwent F-V-profile-based training, and in horizontal variables for those trained based on CODD180°. Furthermore, (D) significant changes in vertical jump performance occurred earlier (at week 4) than those observed in linear and multidirectional speed. Finally, (E) it is worth noting that specific basketball training without individualisation was insufficient for enhancing physical performance, as over 60% of players in this group were classified as NRs. Thus, it is recommended that strength and conditioning coaches implement individualised training stimuli, tailoring programmes to optimise each player’s responsiveness to specific training variables.

According to the principle of specificity and transfer of training [30], previous research [31,32] highlights the importance of training specific force orientation to the ability to be improved. However, recent meta-analysis studies [33] suggest no substantial difference between force-oriented training in enhancing vertical performance, implying that both orientations appear to be effective for the development of vertical actions, such as vertical jumps. Furthermore, horizontally oriented resistance or plyometric training may be superior at enhancing horizontal performance, such as horizontal jumps, sprint acceleration capacity, and change of direction speed [33,34]. Conversely, other meta-analysis studies found that vertically oriented training was more effective for improving performance in horizontal speed and COD actions in basketball players [13]. It is evident that there is still much controversy about how the force orientation of training influences performance in a multidirectional sport such as basketball. This lack of agreement may stem from variations in training protocols, including differences in volume, intensity, and individual player needs [13]. In line with this, studies that have considered additional factors influencing training adaptations suggest that vertical- or horizontal-force-oriented training will improve performance within tasks aligned to the same force orientation in basketball [35] and football players [36]. The current study’s findings confirmed this previous evidence, underscoring the importance of force orientation in training. Specifically, our results demonstrated that each training group achieved the greatest improvements in tasks specific to the trained force orientation, suggesting that training specificity likely facilitates greater neuromuscular coordination and adaptations [37].

To date, the scientific literature has not previously addressed the training individualisation of basketball players. Accordingly, previous meta-analysis studies [13] criticise the lack of intervention studies that individualise training programmes to enhance basketball players’ performance. Non-individualised training protocols may provide insufficient training stimuli to generate adaptations in players’ performance [13]. Supporting this, a recent study examining the effects of combined, non-individualised vertical and horizontal strength training in basketball players [38] reported no significant improvements in vertical jump performance (≤4.28%, *g* ≤ 0.68; *p* ≥ 0.06) or COD actions (≤1.84%, *g* ≤ 0.49; *p* ≥ 0.65) following 8 weeks of training. In contrast, our study observed greater improvements in jump (≥5.62%) and speed (≥1.00%) performance in both force orientation groups with only four weeks of individualised training. Both force orientation groups demonstrated further significant performance gains following eight weeks of intervention (η_p_^2^ ≥ 0.25, *p* < 0.01). Given that both studies involved basketball players, the large differences in training outcomes suggests that individualised training, tailored to each player’s needs, yields superior adaptation effects. Additionally, other authors who analysed the effect of individualised strength training based on the dynamic strength index [39] or the F-V profile [40] on basketball players reported notable performance improvements in basketball players. These findings align with those of the present study, which indicates that individualising training to target specific force orientations leads to better outcomes than generalised basketball training. Moreover, our analysis of NRs revealed that both individualised training protocols resulted in a higher proportion of players responding positively to the training (78% Rs), compared to the non-individualised, basketball-specific group (36% Rs). This evidence suggests that individualised training approaches not only achieve faster and more substantial performance improvements, but also result in fewer NRs.

It is well documented that a short-term intervention (4 to 8 weeks) involving strength or ballistic training programmes can significantly enhance physical performance in male team sport players [39,41]. Regardless of the force orientation trained, male youth team sports players demonstrate improvements in jumping and COD performance within four weeks of training [41]. Similarly, Pleša et al. [39] reported significant gains in jump and COD performance among highly trained basketball players after just five weeks of individualised training based on the dynamic strength index. Thus, these findings suggest that measurable performance improvements can occur within the first month of training. The results of the present study partially align with these findings. Significant improvements were observed in vertical jump actions (≥7.29%, *d* ≥ 1.47, *p* < 0.01) after four weeks of individualised training. However, linear and COD performance did not show significant changes at this time point, although a tendency towards improvement was evident (≥1.00%, *d* ≥ 0.23, *p* ≥ 0.32). Therefore, the timing of performance improvements depends on the specific skill being targeted. In this sense, performance improvements in jumping actions occurred earlier than in speed and COD actions. This discrepancy may be attributed to the complexity of COD movements, which integrate multiple abilities—such as acceleration, deceleration, and re-acceleration—while involving interactions across various force orientations [42]. Consequently, longer training durations, such as that of eight weeks, may be required to elicit significant improvements in COD performance [40], compared to vertical jump, which responded significantly to just four weeks of training. These findings are consistent with a recent meta-analysis on combined training effects on COD speed, which concluded that a minimum intervention period of six to eight weeks is necessary to achieve significant improvements in performance [10].

Despite the novelty of this study, several limitations should be considered when interpreting the results. First, the absence of training load quantification during specific basketball matches and training sessions may have influenced the findings. Nevertheless, all teams included had the same basketball-specific training volume per week. Second, F-V profile assessment could not be carried out with the HG to avoid disturbing their intervention. Nonetheless, this variable was only used to individualise the VG training, so it did not affect the results. Third, the study duration was limited to 8 weeks (16 sessions), which may restrict the generalisability of the findings to adaptations that occur over a full basketball season. Basketball is a sport where players aim to achieve peak performance weekly, and this dynamic could not be evaluated within the scope of the present research. Finally, while individualised training protocols based on the neuromuscular needs of each player were implemented, specific playing positions were not taken into consideration. Therefore, besides designing individualised training programmes, it is recommended that future research should consider the needs of players according to their position.

## 5. Conclusions

These findings highlight the significant potential of programming training based on the individual needs of each player. The ‘one-size-fits-all’ approach appears insufficient to elicit meaningful adaptations in most players. Both individualised training approaches—based on the F-V profile and the CODD180°—proved to be efficient strategies for improving basketball players’ performance. Furthermore, the assessment of these variables is both accessible and time-efficient in a basketball team setting, facilitating the practical implementation of such individualised training programmes within the physical preparation systems used throughout the basketball season. Strength and conditioning basketball coaches are strongly encouraged to consider the specific force orientation targeted in their training designs to optimise player performance. The integration of force-vector-specific training may be critical, as the greatest improvements were achieved in the physical capacities directly aligned with the targeted force vector during the intervention. This underlines the need for specificity in strength training protocols. In summary, addressing the individual needs of each player and ensuring specificity in the development of targeted abilities are key considerations for effective training programme design for basketball players.

## Figures and Tables

**Table 1:** Baseline of the classification and performance variables, inter-group differences, and their respective reliability measures.

Variables	CG (n = 15)	VG (n = 15)	HG (n = 15)	ICC	CV	*p* Value	ES (η_p_^2^)
	Mean (SD)	Mean (SD)	Mean (SD)				
Classification							
F-V Profile	29.1 (34.4)	29.1 (21.6)	-	-	-	0.99	0.01
CODD180°	53.4 (6.58)	51.5 (8.46)	49.6 (6.96)	-	-	0.39	0.04
Performance							
CMJ (cm)	33.7 (4.83)	30.7 (3.02)	34.9 (5.72)	0.99	2.12	0.07	0.12
DJu (cm)	14.2 (3.32)	13.4 (2.88)	16.1 (3.02)	0.98	3.57	0.06	0.12
RSI	0.89 (0.22)	0.67 (0.12) *	0.97 (0.16) *	0.97	4.94	**<0.01**	0.37
LS 10m (s)	1.82 (0.08)	1.86 (0.11)	1.88 (0.13)	0.92	1.81	0.23	0.07
COD45° (s)	1.93 (0.07)	1.97 (0.09)	1.97 (0.09)	0.90	1.12	0.34	0.05
COD90° (s)	2.29 (0.12)	2.33 (0.19)	2.30 (0.12)	0.98	1.33	0.74	0.01
COD180° (s)	2.78 (0.08)	2.81 (0.09)	2.81 (0.14)	0.94	1.65	0.68	0.02
Mean speed (s)	2.20 (0.07)	2.24 (0.10)	2.24 (0.10)	0.93	1.48	0.45	0.04

* Abbreviations: F-V, force–velocity; CODD, change of direction deficit; CMJ, countermovement jump; DJu, unilateral drop jump; cm, centimetres; RSI, reactive strength index; LS, linear speed; COD, change of direction; s, seconds; CG, control group; VG, vertical group; HG, horizontal group; SD, standard deviation; ICC, intraclass correlation coefficient; CV, coefficient of variation; ES, effect size; ηp2, partial eta square. * Significant inter-group differences between VER and HOR (*p* < 0.05). Bold data show statistically significant results (*p* < 0.05).

**Table 2:** Changes in vertical jumping, sprinting, and change of direction performance between pre-(T1), intra-(T2), and post-(T3) intervention for each group.

		T_1_	T_2_	T_3_	% Change		Time	Group × Time
Test	Group	Mean (SD)	Mean (SD)	Mean (SD)	T1−T2	T1−T3	*p* Value (ES)	*p* Value (ES)
Classification variables								
F-V profile	CG	29.1 (34.4)	33.9 (26.1) ^‡^	36.0 (25.3) ^‡^	14.2	19.2	** <0.01 (0.31) **	** <0.01 (0.25) **
	VG	29.1 (21.6) ^a,b^	13.5 (22.9) ^a‡^	10.0 (15.7) ^b‡^	−53.6	−65.6		
	HG	-	-	-	-	-		
CODD 180°	CG	53.4 (6.58)	55.1 (5.91)	55.9 (6.91) ^†^	2.88	2.72	** <0.01 (0.29) **	** <0.01 (0.17) **
	VG	51.5 (8.46)	54.5 (6.22)	51.8 (7.86) *	5.11	−1.09		
	HG	49.6 (6.96) ^b^	47.3 (9.18)	44.5 (6.99) ^b†^*	−9.57	−13.9		
Performance variables								
CMJ (cm)	CG	33.7 (4.83)	32.7 (4.93) ^‡†^	33.5 (5.18) ^‡†^	−3.37	−0.88	0.09 (0.06)	** <0.01 (0.51) **
	VG	30.7 (3.02) ^a,b^	33.2 (3.83) ^a,c‡^	37.2 (4.45) ^b,c‡^*	7.29	17.0		
	HG	34.9 (5.72) ^a,b^	36.9 (7.04) ^a†^	38.5 (7.17) ^b†^*	5.62	9.34		
DJu (cm)	CG	14.2 (3.32)	14.0 (3.08) ^‡^	13.4 (2.93) ^‡†^	−1.61	−5.23	0.56 (0.02)	** <0.01 (0.38) **
	VG	13.4 (2.88) ^a,b^	15.0 (3.29) ^a,c‡^	16.8 (4.22) ^b,c‡^	10.3	18.9		
	HG	16.1 (3.02) ^b^	17.3 (3.89) ^c^	19.0 (3.97) ^b,c†^	6.51	14.6		
RSI	CG	0.89 (0.22)	0.78 (0.20) ^†^	0.77 (0.18) ^‡†^	−16.2	−16.1	** 0.03 (0.09) **	** <0.01 (0.24) **
	VG	0.67 (0.12)	0.73 (0.14)	0.80 (0.14) ^‡^	6.51	15.4		
	HG	0.97 (0.16)	1.02 (0.14) ^†^	1.02 (0.22) ^†^	3.95	2.89		
LS 10 m (s)	CG	1.82 (0.08)	1.80 (0.06)	1.82 (0.11)	−1.05	−0.29	** <0.01 (0.29) **	0.07 (0.10)
	VG	1.86 (0.11) ^b^	1.82 (0.09)	1.79 (0.08) ^b^	−2.25	−4.13		
	HG	1.88 (0.13) ^b^	1.84 (0.09)	1.80 (0.09) ^b^	−2.16	−4.58		
COD 45° (s)	CG	1.93 (0.07)	1.94 (0.14)	1.91 (0.12) ^†^	0.57	−1.39	** 0.04 (0.07) **	0.05 (0.11)
	VG	1.97 (0.09) ^b^	1.94 (0.09) ^c^	1.87 (0.08) ^b,c^	−1.69	−5.61		
	HG	1.97 (0.09) ^b^	1.92 (0.08) ^c^	1.84 (0.07) ^b,c†^	−2.18	−6.37		
COD 90° (s)	CG	2.29 (0.12)	2.37 (0.20)	2.35 (0.14) ^†^	3.03	2.20	** <0.01 (0.17) **	** 0.03 (0.12) **
	VG	2.33 (0.19)	2.34 (0.12)	2.32 (0.13)	0.37	−0.54		
	HG	2.30 (0.12)	2.29 (0.13)	2.24 (0.13) ^†^	−0.97	−3.43		
COD 180° (s)	CG	2.78 (0.08)	2.79 (0.14)	2.82 (0.12) ^†^	0.08	1.33	** <0.01 (0.13) **	** <0.01 (0.26) **
	VG	2.81 (0.09)	2.79 (0.09)	2.72 (0.09)	−0.85	−3.40		
	HG	2.81 (0.14) ^b^	2.72 (0.16) ^c^	2.63 (0.15) ^b,c†^	−3.88	−7.47		
Mean speed (s)	CG	2.20 (0.07)	2.23 (0.13)	2.22 (0.11) ^‡†^	0.77	0.69	** 0.03 (0.08) **	** <0.01 (0.23) **
	VG	2.24 (0.10) ^b^	2.22 (0.08)	2.17 (0.08) ^b‡^	−1.00	−3.27		
	HG	2.24 (0.10) ^b^	2.20 (0.09) ^c^	2.13 (0.09) ^b,c†^	−2.30	−5.49		

* Abbreviations: F-V, force−velocity; CODD, change of direction deficit; CMJ, countermovement jump; DJu, unilateral drop jump; cm, centimetres; RSI, reactive strength index; LS, linear speed; COD, change of direction; s, seconds; CG, control group; VG, vertical group; HG, horizontal group; SD, standard deviation; T1, pre-intervention assessment; T2, intra-intervention assessment; T3, post-intervention assessment; ES, effect size (η_p_^2^, partial eta square). The model was adjusted by inter-group differences, T1. Significant inter-group differences: ^‡^ (between CON and VER; *p* < 0.05), ^†^ (between CON and HOR; *p* < 0.05), * (between VER and HOR; *p* < 0.05). Significant intra-groups differences: ^a^ (between T1 and T2; *p* < 0.05), ^b^ (between T1 and T3; *p* <0.05), ^c^ (between T2 and T3; *p* < 0.05). Bold data show statistically significant results (*p* < 0.05).

**Table 3:** Individual patterns of response on the performance variables by control, vertical, and horizontal groups.

Tests	Individual Response															NRs (%)
	1	2	3	4	5	6	7	8	9	10	11	12	13	14	15	
CONTROL																64%
CMJ	↓	↑	↔	↓	↔	↔	↓	↓	↑	↓	↓	↓	↓	↑	↔	80%
DJu	↔	↑	↑	↑	↑	↑	↑	↑	↑	↑	↔	↔	↔	↔	↔	40%
RSI	↔	↑	↑	↔	↑	↑	↑	↑	↑	↑	↑	↔	↔	↑	↔	33%
LS 10m	↑	↑	↑	↑	↑	↑	↓	↔	↓	↓	↓	↔	↑	↓	↓	53%
COD 45°	↓	↑	↑	↓	↑	↑	↓	↓	↔	↓	↔	↔	↑	↔	↓	67%
COD 90°	↓	↓	↑	↓	↓	↔	↓	↓	↓	↓	↓	↔	↑	↔	↓	87%
COD 180°	↓	↔	↔	↓	↓	↔	↓	↓	↓	↓	↓	↔	↑	↑	↓	87%
VERTICAL																22%
CMJ	↑	↑	↑	↑	↑	↑	↑	↑	↑	↑	↑	↑	↑	↑	↑	00%
DJu	↑	↔	↔	↑	↔	↑	↑	↔	↑	↑	↔	↔	↑	↑	↔	47%
RSI	↑	↔	↔	↔	↑	↑	↑	↑	↑	↔	↑	↑	↑	↑	↑	26%
LS 10m	↑	↑	↑	↑	↑	↑	↑	↑	↑	↑	↑	↑	↑	↔	↑	07%
COD 45°	↑	↑	↑	↑	↑	↑	↑	↑	↑	↑	↑	↑	↑	↔	↑	07%
COD 90°	↓	↑	↔	↑	↑	↔	↑	↑	↑	↑	↓	↑	↓	↓	↔	47%
COD 180°	↑	↑	↔	↔	↑	↑	↔	↑	↑	↑	↑	↑	↑	↑	↑	20%
HORIZONTAL																22%
CMJ	↑	↔	↔	↑	↑	↑	↑	↓	↓	↑	↔	↑	↑	↑	↑	33%
DJu	↑	↔	↔	↔	↑	↑	↑	↑	↑	↑	↑	↑	↑	↔	↑	27%
RSI	↑	↔	↑	↑	↑	↑	↑	↑	↑	↑	↑	↑	↑	↑	↑	07%
LS 10m	↑	↑	↑	↑	↑	↑	↑	↑	↑	↑	↑	↑	↔	↑	↑	07%
COD 45°	↔	↑	↑	↑	↑	↑	↔	↑	↔	↑	↔	↑	↑	↑	↑	27%
COD 90°	↑	↔	↔	↑	↑	↑	↓	↓	↑	↑	↓	↓	↑	↔	↑	47%
COD 180°	↑	↔	↑	↑	↑	↑	↑	↑	↑	↑	↑	↑	↑	↑	↑	07%

Abbreviations: CMJ, countermovement jump; DJu, unilateral drop jump; RSI, reactive strength index; LS, linear speed; m, metres; COD, change of direction; NRs, non-responders. ↑ denote responders (white boxes); ↔ denote non-responders (light grey boxes), and ↓ indicate adverse responses (dark grey boxes). The percentage of participants demonstrate non-responders, including both non- and adverse responses.

## Data Availability

The data that support the findings of this study are available from the corresponding author, F.J.B.-D., upon reasonable request.

## Supplementary Materials

The following supporting information can be downloaded at: https://www.mdpi.com/article/10.3390/sports13070214/s1, Table S1: Vertical individualised training programmes based on the F-V profile for each imbalance threshold. Adapted from Jimenez-Reyes et al. [19].; Table S2: Horizontal individualised training programmes according to the CODD180° thresholds. Based on Barrera-Domínguez et al. [16].

## Abbreviations

The following abbreviations are used in this manuscript:

CMJ	Countermovement jump
COD	Change of direction
CODD	Change of direction deficit
DJu	Unilateral drop jump
LS	Linear sprint
NRs	Non-responders
Rs	Responders
