# Supplementary material for: Effect of Different Individualised Strength Training Approaches to Improve Physical Performance in Male Basketball Players
Source: Sports (Basel). 2025 Jul 2;13(7):214. doi: 10.3390/sports13070214 (PMC12300547; doi:10.3390/sports13070214)
Supplement: Supplementary file 1 [file sports-13-00214-s001.zip › sports-3663978-supplementary.pdf]

Table S1. Vertical individualised training programs based on the F-V for each imbalance threshold. Adapted from Jimenez-Reyes et al. [19].

**Individualised vertical training** (% F-Vimbalance)	**Session**	**Exercise**	**Training load**	**Execution speed**
**High force deficit** (>40% of optimal thresholds)	1	Back squat	80-90% RM	0.68 – 0.51 $m \cdot s^{-1}$ (~10% VL)
		Deadlift trap bar	70-80% RM	0.71 – 0.58 $m \cdot s^{-1}$ (~10% VL)
		SL CMJ	10% BW	
	2	Deadlift barbell	85-95% RM	0.52 – 0.39 $m \cdot s^{-1}$ (~10% VL)
		Clean pull	80% RM	> 1.11 $m \cdot s^{-1}$ (~5% VL)
		CMJ trap bar	80% BW	
**Low force deficit** (between 10% and 40% of optimal thresholds)	1	Back squat	80-90% RM	0.68 – 0.51 $m \cdot s^{-1}$ (~10% VL)
		CMJ trap bar	80% BW	
		SL CMJ	10% BW	
	2	Deadlift barbell	85-95% RM	0.52 – 0.39 $m \cdot s^{-1}$ (~10% VL)
		Clean pull	80% RM	> 1.11 $m \cdot s^{-1}$ (~5% VL)
		SL SJ	BW	
**Well-balanced** (between -10% and 10% of optimal thresholds)	1	Back squat	80-90% RM	0.68 – 0.51 $m \cdot s^{-1}$ (~10% VL)
		Depth jump	BW (30 cm)	
		SL CMJ	10% BW	
	2	Deadlift trap bar	70-80% RM	0.71 – 0.58 $m \cdot s^{-1}$ (~10% VL)
		Clean pull jump	65% RM	
		Abalakov jump	BW	
**Low velocity deficit** (between -10% and -40% of optimal thresholds)	1	Depth jump	BW (30 cm)	
		SL SJ	BW	
		Band assisted CMJ	<BW	
	2	Clean pull jump	65% RM	
		SJ	BW	
		Abalakov jump	BW	
**High velocity déficit** (>40% of optimal thresholds)	1	Band assisted CMJ	<BW	
		Box jump	BW (30 cm)	
		SL CMJ	BW	
	2	Abalakov jump	BW	
		CMJ	BW	
		Clean pull jump	50% RM	

Abbreviations: RM, Repetition Maximum; BW, Body Weight; m/s, meters/seconds; CMJ, Countermovement Jump; SL, Single Leg; SJ, Squat Jump; VL, Velocity Loss.

Additional information:

1. For optimal control of velocity area, intensity and intra-sets volume of training, the execution speed of the strength exercises was monitored with a linear velocity transducer (Vitruve^{TM}, Madrid, Spain), since it has shown to be the most effective control method.
2. Regarding the intra-set volume of the plyometric exercises, a low volume was sought (<60 jumps per session), to maintain the highest possible intensity at all times and cause minimal fatigue.

Table S2. Horizontal individualised training programs according to the CODD180º thresholds. Based on Barrera-Domínguez et al. [16].

Individualised horizontal training (% CODD180º)	Session	Exercise	Training Volume	Training Load
**Multidirectional Speed Dominant** (CODD180º <43%)	1	Moderate Sled Training	3x 4x 10 metres	30% BM (~15% VL)
		Repeated Broad Bound	3x 2x 6 bounds	20 – 40% BM
		HK Lateral Bound	3x 6 bounds	BM
	2	Heavy Sled Training	3x 5x 5 metres	60% BM (~40% VL)
		Repeated Lateral Bound	3x 2x 6 bounds	20 – 40% BM
		HK Broad Bound	3x 6 bounds	BM
**Balanced** (CODD180º between 43% and 49%)	1	Technical: 5m Acc to Dec	3x 4 reps EL	BM
		FW Forward Lunge	3x 2+6 reps EL	FW
		AEC Lateral Lunge	3x 6 reps EL	Bands AEC
	2	Heavy Sled Training	3x 5x 5 metres	60% BM (~40% VL)
		Repeated Lateral Bound	3x 2x 6 bounds	20 – 40% BM
		HK Broad Bound	3x 6 bounds	BM
**Linear Speed Dominant** (CODD180º >49%)	1	Technical: 5m Acc to Dec	3x 4 reps EL	BM
		FW Forward Lunge	3x 2+6 reps EL	FW
		AEC Lateral Lunge	3x 6 reps EL	Bands AEC
	2	Technical: COD180º to Dec	3x 2 reps EL	BM
		FK Lateral Lunge	3x 2+6 reps EL	FW
		AEC Forward Lunge	3x 6 reps EL	Bands AEC

Abbreviations: CODD, Change of Direction Deficit; HK, Half-Kneeling; Acc, Acceleration; Dec, Deceleration; FW, Flywheel; AEC, Accelerated Eccentric Loading; COD, Change of Direction; EL, Each limb; BM, Body Mass; VL, Velocity Loss.

Additional information:

1. Resistance sprint training was undertaken with a pulley system (Exer-Genie, Thousand Oaks, CA, USA) at moderate (30-40% body mass) and high (60-70% body mass) intensities with short distances (5-10 metres) to emphasise the acceleration phase.
2. In exercises with flywheel, two preparatory reps were performed in each set prior to the six targeted reps to ensure effectiveness; and players were instructed to slow down the movement in the last third of the eccentric phase and exit the "hole" as quickly as possible to ensure a true eccentric overload.
3. Regarding the volume of the accelerated eccentric loading and plyometric exercises, it was low (<60 contacts per session), to maintain the highest possible intensity at all times and cause minimal fatigue.
4. To work and improve COD technique, exercises involving the action itself were performed with instructions such as (i) “slam on the brakes and drop the hips”; (ii) “cushion and push/punch the ground away”; and (iii) “face towards the direction of travel”.
